# The endocannabinoid system as a possible target to treat both the cognitive and emotional features of post-traumatic stress disorder (PTSD)

**DOI:** 10.3389/fnbeh.2013.00100

**Published:** 2013-08-09

**Authors:** Viviana Trezza, Patrizia Campolongo

**Affiliations:** ^1^Department of Sciences, Section of Biomedical Sciences and Technologies, University “Roma Tre,”Rome, Italy; ^2^Department of Physiology and Pharmacology, Sapienza University of RomeRome, Italy

**Keywords:** endocannabinoids, cannabis, memory, anxiety, trauma exposure

## Abstract

Post-traumatic stress disorder (PTSD) is a psychiatric disorder of significant prevalence and morbidity, whose pathogenesis relies on paradoxical changes of emotional memory processing. An ideal treatment would be a drug able to block the pathological over-consolidation and continuous retrieval of the traumatic event, while enhancing its extinction and reducing the anxiety symptoms. While the latter benefit from antidepressant medications, no drug is available to control the cognitive symptomatology. Endocannabinoids regulate affective states and participate in memory consolidation, retrieval, and extinction. Clinical findings showing a relationship between Cannabis use and PTSD, as well as changes in endocannabinoid activity in PTSD patients, further suggest the existence of a link between endocannabinoids and maladaptive brain changes after trauma exposure. Along these lines, we suggest that endocannabinoid degradation inhibitors may be an ideal therapeutic approach to simultaneously treat the emotional and cognitive features of PTSD, avoiding the unwanted psychotropic effects of compounds directly binding cannabinoid receptors.

Post-traumatic stress disorder (PTSD) is a psychiatric disorder of significant prevalence and morbidity (Layton and Krikorian, 2002). In the overall population, more than two thirds of persons may experience a serious traumatic event at some point in lifetime (Javidi and Yadollahie, 2012). Although not everyone develops PTSD after experiencing a traumatic event, the lifetime prevalence of PTSD is high, being estimated as 8.2% in Europe and in the United States, up to 9.2% in Canada (Kessler et al., 1995; Darves-Bornoz et al., 2008; Van Ameringen et al., 2008). More than a third of PTSD patients fail to recover even after many years of treatment (Darves-Bornoz et al., 2008), showing a significant impairments in many aspects of health-related quality of life, including psychosocial functioning (Schelling et al., 1998).

Feeling afraid is a natural response to threats and triggers many physiological changes to prepare the body to defend against the danger or to avoid it. In PTSD, this reaction is changed or damaged. Even if anxiety is a common symptom of PTSD patients, the pathogenesis of the disorder relies on paradoxical changes of memory processing (Cohen et al., 2006; Parsons and Ressler, 2013). From a physiological point of view, memories characterized by a strong emotional salience tend to be well consolidated, they are often retrieved in our brain and therefore tend not to be extinct; from an evolutionary perspective, this is of crucial importance for survival. However, in PTSD patients, all or part of this processes may become maladaptive. Three symptom categories characterize the disorder: (1) persistent re-experience of the traumatic event; (2) persistent symptoms of increased arousal; and (3) persistent avoidance of stimuli associated with the trauma, which may include amnesia for important aspects of the traumatic event (Brewin, 2001). These symptoms reflect excessive retrieval of traumatic memories that are again consolidated, thus cementing the traumatic memory trace, and retaining its vividness and power to evoke distress for decades or even a lifetime (de Quervain et al., 2009). It appears from this symptomatology that three phases of memory processing may become maladaptive and of crucial importance in the development and maintenance of PTSD: consolidation, retrieval, and extinction.

PTSD is heterogeneous in its nature, and often associated with other psychiatric comorbidities; for these reasons, treating PTSD is rather difficult, and the disorder may persist over the patient's lifetime (Albucher and Liberzon, 2002). The therapeutic options to treat the anxiety symptoms of PTSD currently include serotonin reuptake inhibitors (SSRIs), serotonin–norepinephrine reuptake inhibitors (SNRIs), tricyclic antidepressants (TCAs), monoamine oxidase inhibitors (MAOi), anticonvulsants, atypical antipsychotics and benzodiazepines (Albucher and Liberzon, 2002). Although SSRIs emerge as the preferred first line treatment to treat the anxiety symptoms of PTSD (Dow and Kline, 1997; Ipser et al., 2006), a large proportion of patients fails to respond to these medications (Ipser et al., 2006). Furthermore, no suitable treatment is currently available to treat the maladaptive cognitive features of PTSD and/or to prevent its development. This limitation is due to the scarce knowledge of PTSD neurobiology that hampers the identification of new pharmacological targets to treat this disorder. As Albucher and Liberzon (2002) pointed out, the diversity of the symptoms such as flashbacks, nightmares, hyperarousal, avoidance, numbing, anxiety, anger, impulsivity, or aggression suggests the involvement of multiple neurotransmitter systems (Goodman et al., 2012; Packard and Goodman, 2012).

An ideal pharmacological treatment for PTSD would be a drug able to block the pathological over consolidation and continuous retrieval of the traumatic event, while enhancing its extinction and reducing the anxiety symptoms. Although no such drug is currently available, recent clinical (Fraser, 2009; Hauer et al., 2013; Neumeister et al., 2013) and preclinical (Lutz, 2007; Akirav, 2011; Berardi et al., 2012; Ganon-Elazar and Akirav, 2012) studies point to the endocannabinoid system as a possible ideal therapeutic target to treat both the emotional and cognitive dysfunctions characterizing PTSD (Neumeister, 2013).

The central endocannabinoid system is a neuroactive lipid signaling system in the brain which shows functional activity since early stages of brain development; by controlling neurotransmitter release, it plays a relevant role in brain function during both pre- and post-natal life (Fernandez-Ruiz et al., 2000; Harkany et al., 2007; Trezza et al., 2008; Campolongo et al., 2009b, 2011). The endocannabinoid system consists of cannabinoid receptors (CB1 and CB2), their endogenous lipid ligands (endocannabinoids) and the enzymatic machinery for endocannabinoid synthesis and degradation (Piomelli, 2003; Di Marzo et al., 2005). Due to the wide expression of cannabinoid receptors throughout limbic regions of the brain, endocannabinoids control both emotional behavior and cognitive processes (Riedel and Davies, 2005; Campolongo et al., 2007, 2009a; Hill and Gorzalka, 2009; Atsak et al., 2012; Campolongo et al., 2012). Thus, while preclinical studies assessing the consequences of cannabinoid receptor blockade or activation on emotional responses have yielded sometimes controversial results, consensus exists that endocannabinoids have an essential role in maintaining emotional homeostasis (Haller et al., 2002, 2004; Hill and Gorzalka, 2009; Moreira and Wotjak, 2010; Parolaro et al., 2010; Ruehle et al., 2012). Similarly, evidence exists that administration of cannabinoid drugs in animals influences memory consolidation, retrieval and extinction (Marsicano et al., 2002; Niyuhire et al., 2007; Marsicano and Lafenetre, 2009; Atsak et al., 2012; Campolongo et al., 2013). In particular, systemic administration of cannabinoid agonists impairs memory retrieval (Niyuhire et al., 2007) while facilitating memory extinction (Lutz, 2007). Direct evidence has been provided that endocannabinoids modulate emotional memory processing acting in the basolateral complex of the amygdala (BLA), in the hippocampus (de Oliveira Alvares et al., 2005, 2008; Campolongo et al., 2009a; Atsak et al., 2012) and in the prefrontal cortex (Egerton et al., 2006), key brain regions involved in memory consolidation, retrieval and extinction of emotionally arousing experiences (McGaugh, 2004; Quirk and Mueller, 2008; Roozendaal et al., 2008; Herry et al., 2010), and dysfunctional in PTSD patients (Bremner et al., 2008; Hughes and Shin, 2011). Interestingly, emerging empirical work has indicated a link between traumatic event exposure and cannabis use. Data from the National Comorbidity Study demonstrated that adults suffering from PTSD were three times more likely to have cannabis dependence as compared with those without PTSD (Kessler et al., 1995). Studies involving military veterans have demonstrated an even higher rate of cannabis abuse among military veterans with PTSD (Stewart et al., 1998; Bonn-Miller et al., 2011). A positive association between PTSD and cannabis use among teenagers has also been reported (Cornelius et al., 2010). These results could be partially explained by recent data demonstrating that PTSD patients present important changes of plasma endocannabinoid levels and elevation in amygdala-hippocampal-cortico-striatal CB1 receptor availability (Hauer et al., 2013; Neumeister, 2013; Neumeister et al., 2013). The comorbidity between cannabis abuse and PTSD is always described in literature as a negative aspect, with the increase in substance abuse after a disaster as a cause for public long-term health consequences. However, another side of the coin needs to be considered. It is possible that PTSD patients use cannabis as a self-medication. In support of this hypothesis, one study among Vietnam veterans indicated that cannabis use was helpful in managing PTSD symptoms, with particular respect to the hyperarousal state (Bremner et al., 1996). It has been shown that there is a correlation between post-traumatic stress symptom severity and motivation to use marijuana in order to cope with emotional distress (Bonn-Miller et al., 2007). Although the majority of the currently available clinical studies highlights the beneficial effects of cannabis use in PTSD patients, the positive association between cannabis use and relief from PTSD symptoms is not an universal finding. Thus, it has also been documented that, in certain conditions, cannabis abuse may facilitate PTSD development (Cougle et al., 2011). This may be due to the fact that direct activation of cannabinoid receptors by the active ingredient of cannabis Delta-9-tetrahydrocannabinol leads to a rapid downregulation of the endocannabinoid signaling system (Hirvonen et al., 2012), resulting in tolerance. The complex scenario that emerges from the clinical setting makes it difficult to draw final conclusions about the relationship between cannabis use and PTSD. Preclinical studies allow to control for the confounding variables that characterize the clinical observations, and therefore can provide essential information to elucidate the link between endocannabinoids and emotional memory processing, from physiological to pathological conditions. Thus, as highlighted above, it has been demonstrated that cannabinoid compounds strongly facilitate memory extinction in animals (Marsicano et al., 2002; Lutz, 2007), while impairing memory retrieval (Niyuhire et al., 2007; Atsak et al., 2012). It is thus tempting to speculate that cannabinoid compounds can attenuate the excessive retrieval of the traumatic event experienced by PTSD patients, while facilitating its extinction. Memory consolidation for emotionally salient events is also affected by cannabinoid drugs, although the results of the preclinical studies performed so far are controversial. Thus, it has been shown that post-training administration of cannabinoid receptor direct or indirect agonists facilitates memory consolidation in the inhibitory avoidance task (Campolongo et al., 2009a; Hauer et al., 2011). These findings suggest that activation of cannabinoid receptors shortly after experiencing a stressful event could facilitate the development of maladaptive memories of this event. This, in turn, may provide preclinical rationale to the finding that the use of drugs indirectly enhancing endocannabinoid activity, such as propofol, or the use/abuse of cannabis, shortly after the experience of an aversive event, may facilitate PTSD development in humans and has to be avoided in the aftermath of an aversive experience (Cougle et al., 2011; Hemmings and Mackie, 2011; Usuki et al., 2012). However, cannabinoid agonists administered to rats shortly after exposure to a series of intense stressful events have been reported to prevent the impairment in avoidance extinction induced by the traumatic experience (Ganon-Elazar and Akirav, 2009, 2012, 2013). These findings leave open the possibility that cannabinoid drugs may be good candidates for secondary prevention of PTSD, that is, may be a good therapeutic option immediately after trauma exposure (Zohar et al., 2011). It clearly appears from this scenario that, if from one side the data about the effects of cannabinoid drugs on memory retrieval and extinction are quite consistent and suggest that these compounds may facilitate PTSD recovery, on the other side the role of cannabinoids in memory consolidation is still debated. More research is therefore warranted to determine the extent to which differences in doses, routes of administration, timing of exposure and behavioral tasks used may be responsible for the opposite effects of cannabinoid agonists on memory consolidation reported so far. Conversely, encouraging clinical data exist on the use of cannabinoid compounds after the onset of the pathology (weeks or months after the experience of a traumatic event, when the memory consolidation of the traumatic event is completed) (Passie et al., 2012). A recent clinical trial to evaluate the effects of nabilone, a cannabinoid receptor agonist, on treatment-resistant nightmares in PTSD patients demonstrated that the majority of patients (72%) receiving nabilone experienced either cessation of nightmares or a significant reduction in nightmare intensity (Fraser, 2009). Subjective improvement in sleep time, the quality of sleep, and the reduction of daytime flashbacks were also noted by some patients (Fraser, 2009). This is the first report of the use of nabilone for the management of treatment-resistant nightmares in PTSD. Although this evidence is encouraging, further studies on larger cohorts and with a more accurate identification of possible side effects of chronic use of direct cannabinoid agonists are warranted. The use of drugs that directly bind and activate brain cannabinoid receptors is indeed limited by their abuse potential (Tanda and Goldberg, 2003; Economidou et al., 2007; Ashton, 2012). Two alternative pharmacological approaches exist to target cannabinoid receptors in the brain, without inducing abuse liability (Gobbi et al., 2005; Bortolato et al., 2006; Justinova et al., 2008). First, it has recently been reported that the non-psychotomimetic constituent of cannabis cannabidiol facilitates disruption of contextual fear memories (Stern et al., 2012) in rats while inducing anti-anxiogenic-like effects in rats and humans (Bitencourt et al., 2008; Bergamaschi et al., 2011). Alternatively, several preclinical studies have identified endocannabinoid deactivation inhibitors as a novel therapeutic approach for the treatment of neuropsychiatric disorders. In particular, indirect cannabinoid agonists have been proposed as anxiolitic and antidepressant agents (Kathuria et al., 2003; Bortolato et al., 2006; Piomelli et al., 2006; Vinod and Hungund, 2006) and have been reported to facilitate extinction of fear memory in rodents (Bitencourt et al., 2008; Pamplona et al., 2008). Thus, these compounds may prove effective to ameliorate the anxiety symptoms of PTSD and, at the same time, an increase in the endocannabinoid tone may be useful to treat the cognitive features (Varvel et al., 2007) of the pathology. These dual effects make these drugs gold candidates in the treatment and prevention of PTSD. Much attention, however, has to be dedicated to the time framing of pharmacological treatment, with an attempt to avoid the first early phases of memory consolidation.

It clearly appears that a deeper insight into the role of endocannabinoid neurotransmission in emotional memory processing, both in physiological and pathological conditions, will shed light in the neurobiological basis of PTSD; this, in turn, will open new frontiers for alternative and more efficacious therapeutic approaches for a complete resolution of the pathology.

## Conflict of interest statement

The authors declare that the research was conducted in the absence of any commercial or financial relationships that could be construed as a potential conflict of interest.
